# Supplementary material for: Trajectory inference across multiple conditions with condiments
Source: Nat Commun. 2024 Jan 27;15:833. doi: 10.1038/s41467-024-44823-0 (PMC10821945; doi:10.1038/s41467-024-44823-0)
Supplement: Supplementary file 1 — Supplementary Information [file 41467_2024_44823_MOESM1_ESM.pdf]

S-1 Supplementary methods

S-1.1 Other methods implemented in the **condiments** package to test equality of distributions.

These methods were found to be less efficient in initial benchmarking, but are implemented in case users wish to apply them.

S-1.1.1 Multivariate case: The two-sample kernel test

Mean maximum discrepancy. The two-sample kernel test was defined by Gretton et al. [37] and relies on the mean maximum discrepancy (MMD). Considering a kernel function

$$\begin{aligned} k : \mathbb{R}^d \times \mathbb{R}^d &\to \mathbb{R} \\ (x, y) &\mapsto k(x, y) \end{aligned}$$

the MMD is then defined as

$$MMD^2(\mathbf{P}_1, \mathbf{P}_2, k) \equiv \mathbb{E}_{\mathbf{P}_1,\mathbf{P}_1}[k(X, X')] + \mathbb{E}_{\mathbf{P}_2,\mathbf{P}_2}[k(Y, Y')] - 2\mathbb{E}_{\mathbf{P}_1,\mathbf{P}_2}[k(X, Y)].$$

For a properly defined kernel, we have $MMD^2(\mathbf{P}_1, \mathbf{P}_2, k) = 0$ i.i.f. $\mathbf{P}_1 = \mathbf{P}_2$.

Unbiased statistic. Following Gretton et al. [37], we define the unbiased MMD statistic:

$$MMD_u^2(\mathbf{X}, \mathbf{Y}) \equiv \frac{1}{n(n-1)} \sum_{i=1}^{n} \sum_{j \neq i}^{n} k(\mathbf{X}_i, \mathbf{X}_j) + \frac{1}{m(m-1)} \sum_{i=1}^{m} \sum_{j \neq i}^{m} k(\mathbf{Y}_i, \mathbf{Y}_j) - \frac{2}{mn} \sum_{i=1}^{n} \sum_{j=1}^{m} k(\mathbf{X}_i, \mathbf{Y}_j).$$

Linear statistic for faster computations. While the MMD^2 offers fast convergence, it can be burdensome to compute when m and n get large. Gretton et al. [37] propose a linear statistic in the case $m = n$. We can extend this in the general setting by just sampling a fixed fraction of the terms of each sum. This lowers kernel computation costs drastically.

Null distribution of the statistic. For some kernels, the MMD_u^2 follows some theoretical inequalities under the null that allows one to define rejection regions. However, this is not always the case. Therefore, in practice, we instead rely on permutations to compute a null distribution for the test statistic. Under the null, $\mathbf{X}_i$ and $\mathbf{Y}_j$ are from the same distribution so they can be swapped in the sums. We can therefore generate an empirical distribution and use it to define rejection regions.

S-1.1.2 Multivariate case: Optimal transport

We consider the Wasserstein distance [44, 45], also known as earth's mover distance, between the two distributions, estimated using the samples $\mathbf{X}$ and $\mathbf{Y}$. We can generate a null distribution for this metric by permuting observations in the combined $\mathbf{X}$ and $\mathbf{Y}$ datasets, thereby obtaining a valid test for $H_0 : \mathbf{P}_1 = \mathbf{P}_2$. This works in any number of dimensions, but is limited to the two-sample case.

S-1.1.3 No confounding

Some settings allow for proper experimental design, such that there is no confounding and each batch contains cells from each condition. In such cases, we propose two additional methods.

distinct [46] is a statistical testing method that uses permutation to compare distributions and relies on the existence of multiple batches.

We also implemented a version of the `topologyTest` that is run on each batch separately, using any of the previously mentioned statistical testing methods. The p-values can then either be combined via Stouffer's Z-score method [41], or each batch can be analyzed separately to identify potential normalization problems or interesting biological results.

S-1.2 Mutinomial test

We consider a set of categories arbitrarily numbered from 1 to C. Additionally, we consider a null distribution $\mathbf{C}_0$, defined on 1 to C by a vector of probabilities $\mathbf{p} = \{p_c\}_{c=1}^{C}$. Then, given a set of n i.i.d. realizations $(c_1, \ldots, c_n)$ of a random variable $\mathbf{C}$, we can test the null hypothesis $H_0 : \mathbf{C} \sim \mathbf{C}_0$ or, equivalently, $H_0 : \mathbf{P}(\mathbf{C} = c) = p_c, \forall c \in \{1, \ldots, C\}$ as follows. Under the null, $\mathbf{P}_{H_0}(c_i) = p_{c_i}$ and the associated p-value of the multinomial test can be defined as:

$$P(x, \mathbf{p}) = \sum_{y \in \{1,\ldots,C\}^n : \mathbf{P}_{H_0}(y) \leq \mathbf{P}_{H_0}(x)} \mathbf{P}_{H_0}(y).$$

It verifies: $\forall \alpha \in [0:1], \mathbf{P}_{H_0}(P(x, \mathbf{p}) \leq \alpha) \leq \alpha$.

S-1.3 Case studies: Pre-processing.

TGF-β. The two conditions are normalized separately using SCTransform [47] and then integrated using Seurat [20]. The reduced-dimensional representation is computed using UMAP [28] on the top 50 principal components (PC). The imbalance score is computed with parameters $k = 20$ and $smooth = 40$. The trajectory is estimated using slingshot. The `topologyTest` is run with 100 permutations with the Kolmogorov-Smirnov test and default threshold of .01. The `progressionTest` is run with defaults. All genes with at least 2 reads in 15 cells are kept. The smoothers are fitted for each gene using 7 knots, as recommended by the `evaluateK` function. Gene set enrichment analysis is done using the fgsea [32] package on the GO Biological Process ontology sets.

Fibrosis. The reduced-dimensional coordinates were obtained from the original publication. The imbalance score is computed with parameters $k = 20$ ans $smooth = 30$ and the `topologyTest` is run with the default parameters. The trajectories are estimated using slingshot. The `progressionTest` and `fateSelectionTest` are run with defaults. All genes with at least 2 reads in 30 cells are kept. The smoothers are fitted for each gene using 6 knots, as recommended by the `evaluateK` function.

TCCD. We follow the workflow of the original publication. The dataset is first filtered using the cell type assignments from the original publication to only retains cells labelled as hepatocytes. The count matrix is scaled using Seurat [20] and reduced-dimensional coordinates are computed using UMAP [28] on the top 30 PCs. The imbalance score is computed with default k and $smooth = 5$. The trajectory is estimated using slingshot. The `topologyTest` is run with 100 permutations with the Kolmogorov-Smirnov test and default threshold of .01. The `progressionTest` is run with defaults. All genes with at least 3 reads in 10 cells are kept. The smoothers are fitted for each gene using 7 knots, as recommended by the `evaluateK` function.

KRAS. The reduced-dimensional coordinates were obtained from the original publication, using Zinbwave [48]. The imbalance score is run with defaults and the `topologyTest` is run with 100 permutations with the classifier test and default threshold of .01. The trajectories are estimated using slingshot with parameters $reweight = FALSE$ and $reassign = FALSE$. The `progressionTest` and `fateSelectionTest` are run with defaults. All genes with at least 5 reads in 10 cells are kept. The smoothers are fitted for each gene using 6 knots, as recommended by the `evaluateK` function.

S-1.4 Runtimes

We use the **TGF-β** dataset to compute runtimes as a function of the number of cells. The number of genes is only a factor in Step 3, and can be naively parallelized.

We randomly sample cells, by spatial Id and condition, with $n \in \{100, 200, 500, 1000, 2000, 5000, 9268\}$, 10 times, and we compute the runtimes of each step, varying the number of knots in Step 3.

S-1.5 Statistical validity and properties of the p-values of Step 3

Statistical validity of the p-values : To assess the statistical validity of the p-values produced by the `conditionTest`, we generate p-values under the null. To do so, we use the **TGFB** dataset, but we randomly assign each cell to a condition A or B that we use as input to the `fitGam` function. We then run the `conditionTest`, testing against varying levels of log-fold-change cut-offs. Results are presented in the corresponding supplementary results section.

Controlling the false discovery rate : To assess how the false discovery rate is controlled via Benjamini-Hochberg correction [24], we generate a new simulated dataset. We first use `fitGAM` on all genes of the **TGF-β** dataset, using only the $5,027$ cells from the control condition, and extract the fitted negative binomial distribution. We then sample from that distribution, using the pseudotimes of the original cells as inputs. For half of the cells, and for genes among the top 1,000 most associated with pseudotime via the `associationTest`, we amplify the association by multiplying the coefficients s_{jlc} (see Equation 11) by a factor of 2, meaning that we create a log-fold-change effect of 2 for those genes.

We then reran `fitGAM` on this simulated dataset, followed by the `conditionTest` testing against varying log-fold-change cut-offs. This entire procedure was repeated 10 times. Results are presented in the corresponding supplementary results section.

S-2 Supplementary Results

S-2.1 Examples

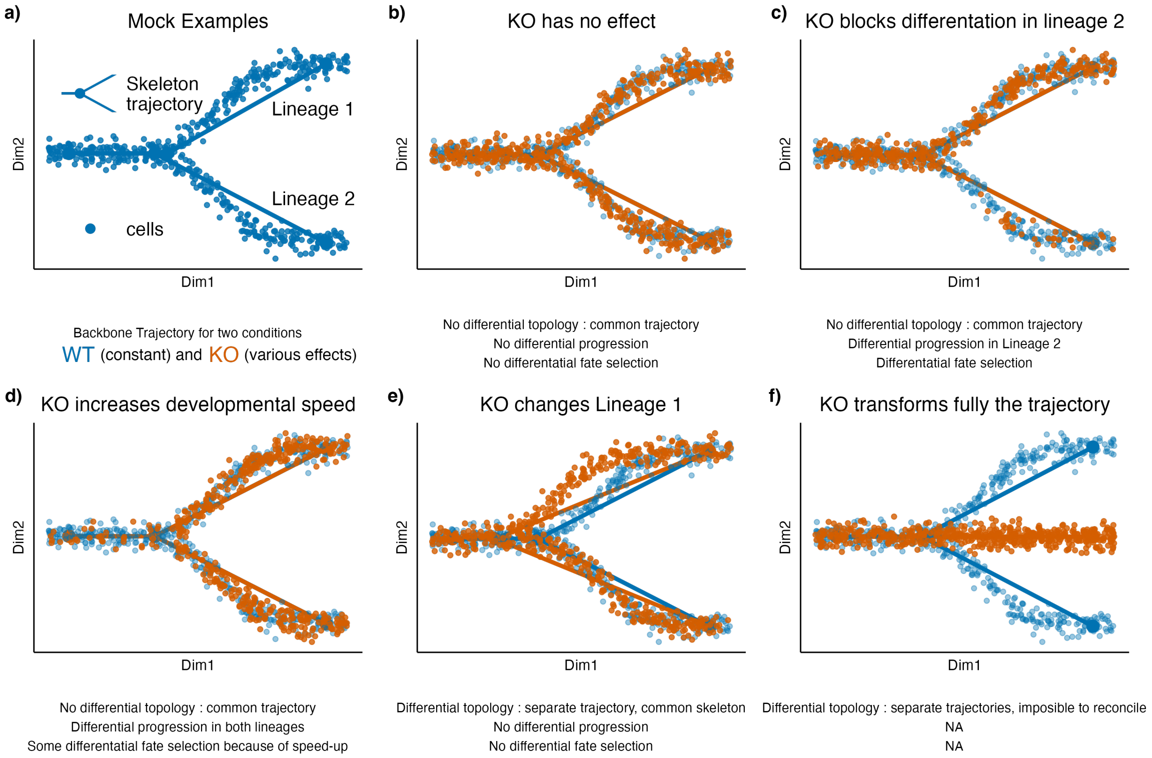

Figure S1: *Illustrating the first two steps of the* condiments *workflow with several scenarios.* **(a)** The examples are all built on a similar wild-type backbone, i.e., two lineages that slowly diverge in the absence of knocking out. Cells either originate from a wild-type (WT, blue) or a knock-out (KO, orange) condition. In **(b)**, the knock-out has no effect, all three tests fail to reject their null hypothesis. In **(c)**, the knock-out partly blocks differentiation along Lineage 2, meaning that fewer cells develop along that lineage. In this case, while the `topologyTest` fails to reject the null, we have both differential progression along Lineage 2 and differential fate selection. In **(d)**, the knock-out speeds development, so there are more orange cells toward the end of both lineages. This leads to both differential progression and fate selection. In **(e)**, the knock-out modifies the intermediate stage for Lineage 1 and changes where the lineages bifurcates; based on the `topologyTest`, we fit one trajectory per condition. However, the skeleton structure is unchanged, so there is a mapping between the two trajectories and we can still test for differential progression and fate selection. In both cases, we fail to reject the null. Finally, in **(f)**, the knock-out fully disrupts the developmental process: all cells in the knock-out condition progress along a new lineage. Here, we fit separate trajectories and these cannot be reconciled easily, so we cannot proceed to Steps 2 and 3.

S-2.2 Runtimes

Step 3 is indeed by far the slowest of all, followed by Step 1. However, note that both Step 1 and 3 can be fully parallelized and can therefore take advantage of multiple cores. Step 3 scales in the number of genes, which can be offset with parallelization, but also with the number of parameters to estimate.

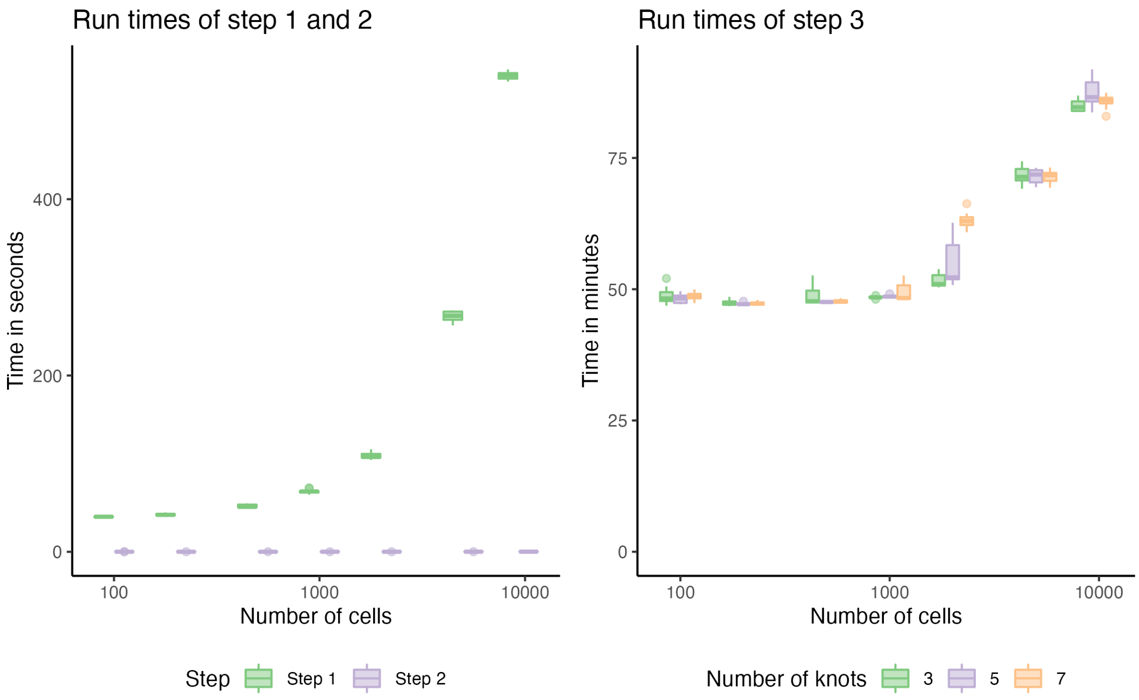

Figure S2: ***TGF-β** dataset: runtimes.* We compute the runtimes of each step, with 10 repeats, and by downsampling the dataset. Step 1 and 3 are the longest, but they can be parallelized.

S-2.3 Simulations for Step 2

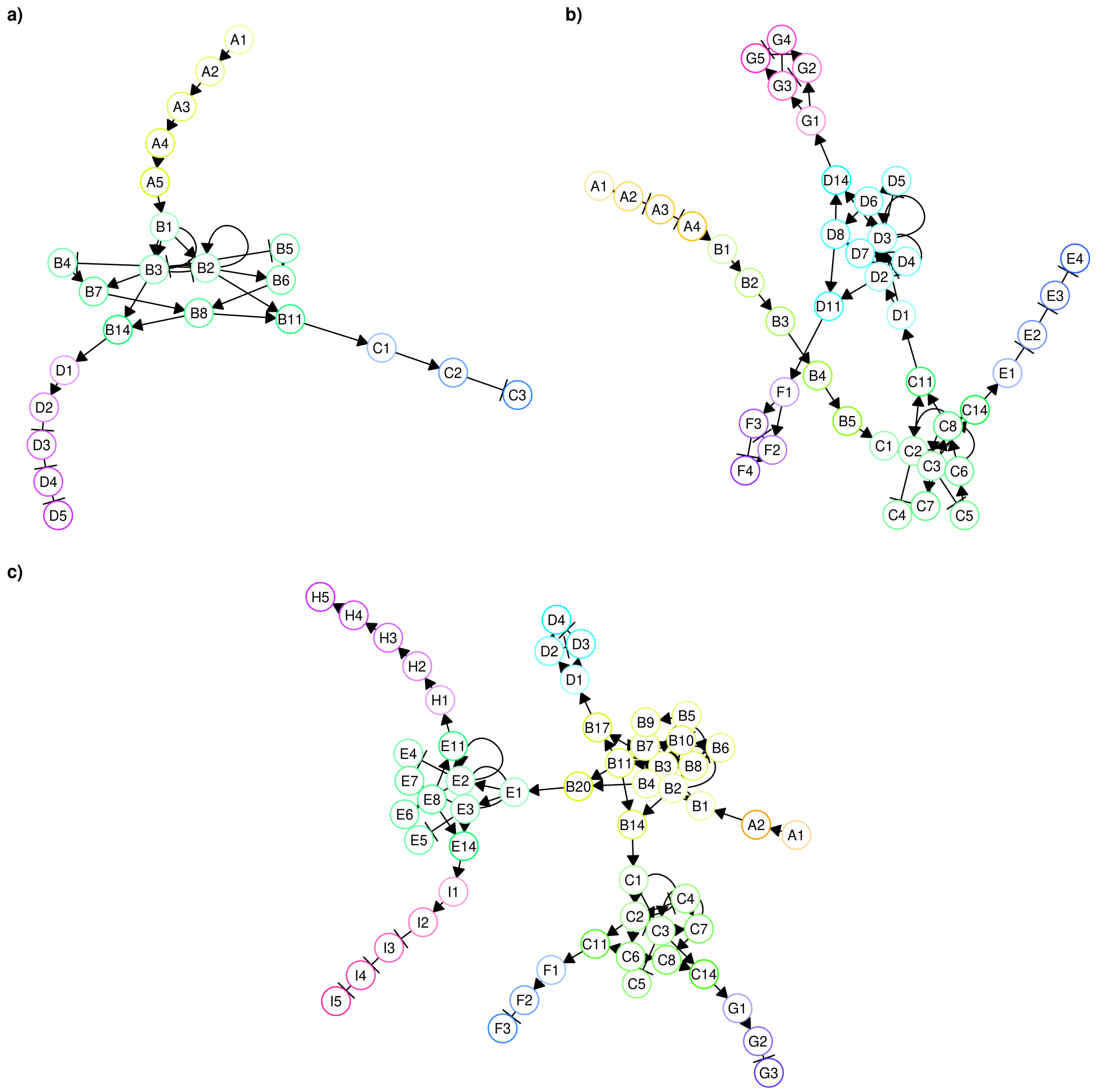

Figure S3: *Simulation example.* Regulator networks for the **(a)** two-lineage, **(b)** three-lineage, and **(c)** five-lineage trajectories.

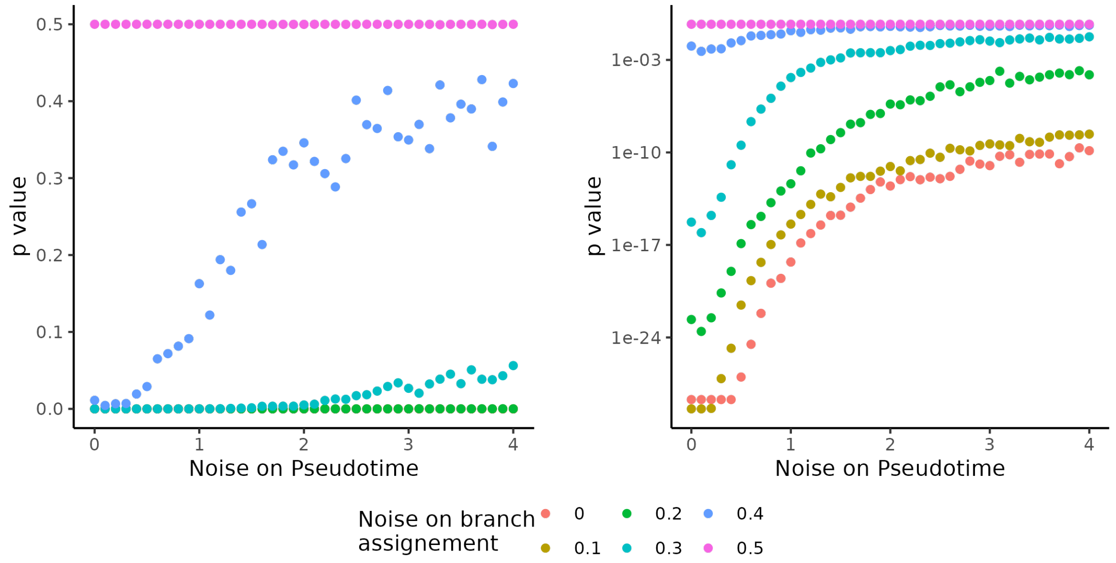

Figure S4: *Sensitivity of Step 2 to incorrect trajectory inference.* The p-value associated with the progressionTest (y-axis, normal scale and log-scale) are displayed for increasing levels of noise on pseudotime estimation (x-axis) and lineage assignment (color). For each noise level, we do 100 repetitions and plot the median p-value.

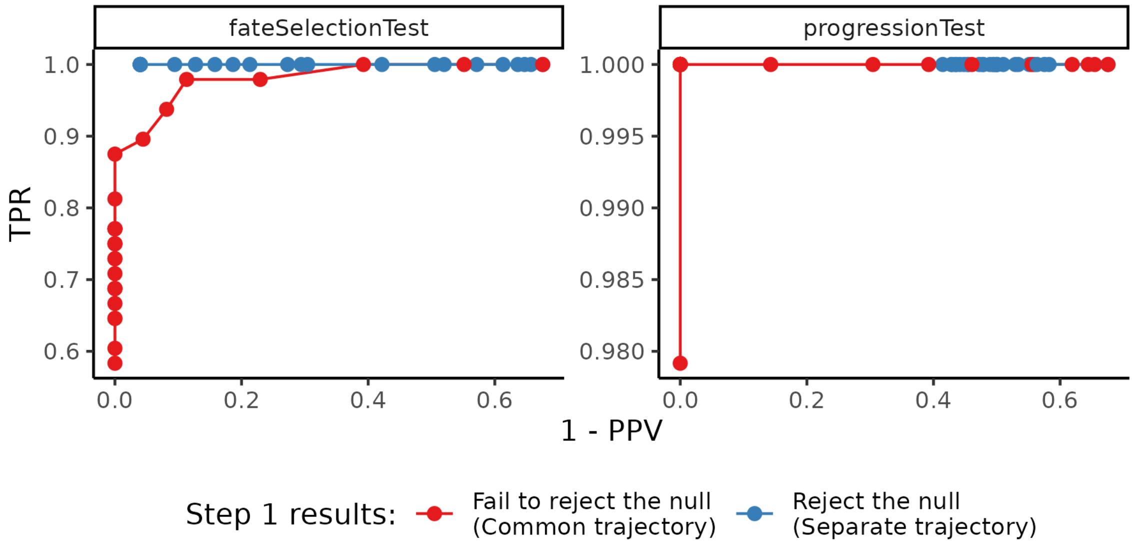

Figure S5: *Sensitivity of Step 2 to incorrect decision at Step 1.* The receiver operating characteristic (ROC) curves are displayed when performing either the `fateSelectionTest` (left panel) or the `progressionTest` (right panel), downstream of the following two choices after Step 1: fitting a common trajectory (in red) and a separate trajectory per condition (in blue). Results are similar, although the `fateSelectionTest` is more impacted

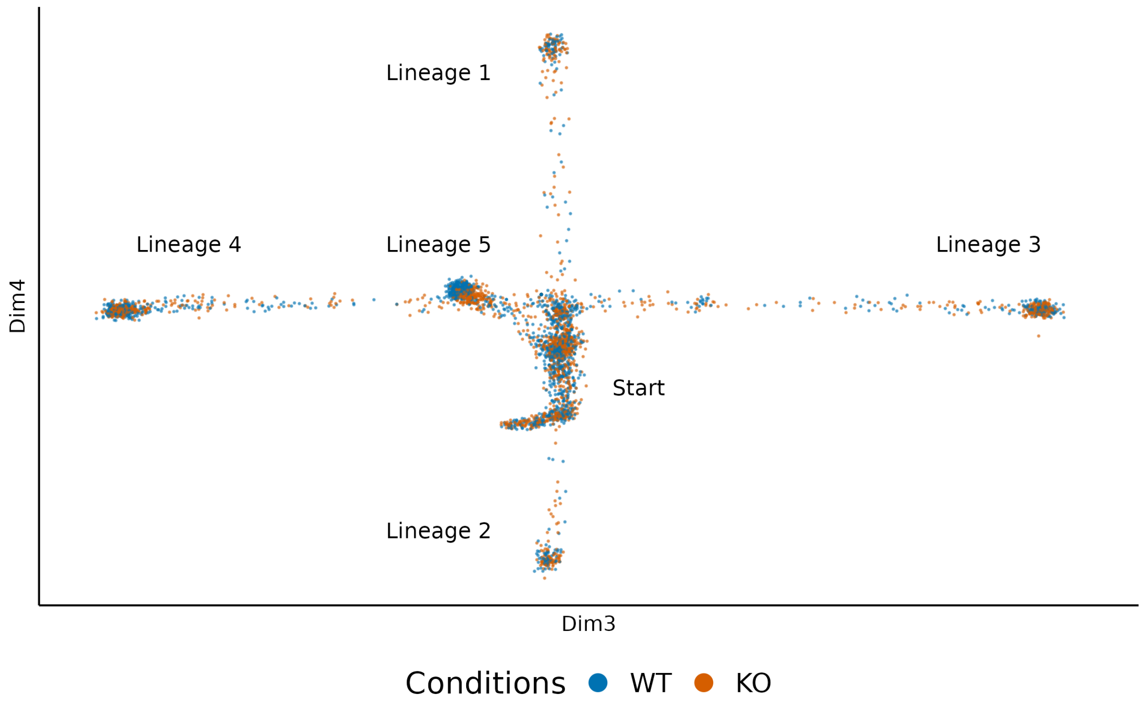

Figure S6: *Simulated dataset of the fourth type* Reduced-dimensional representation of a dataset with five lineages and 3 conditions, such as the one in Fig. 2d, using the third and fourth dimensions to show that the lineages do separate when more dimensions are considered.

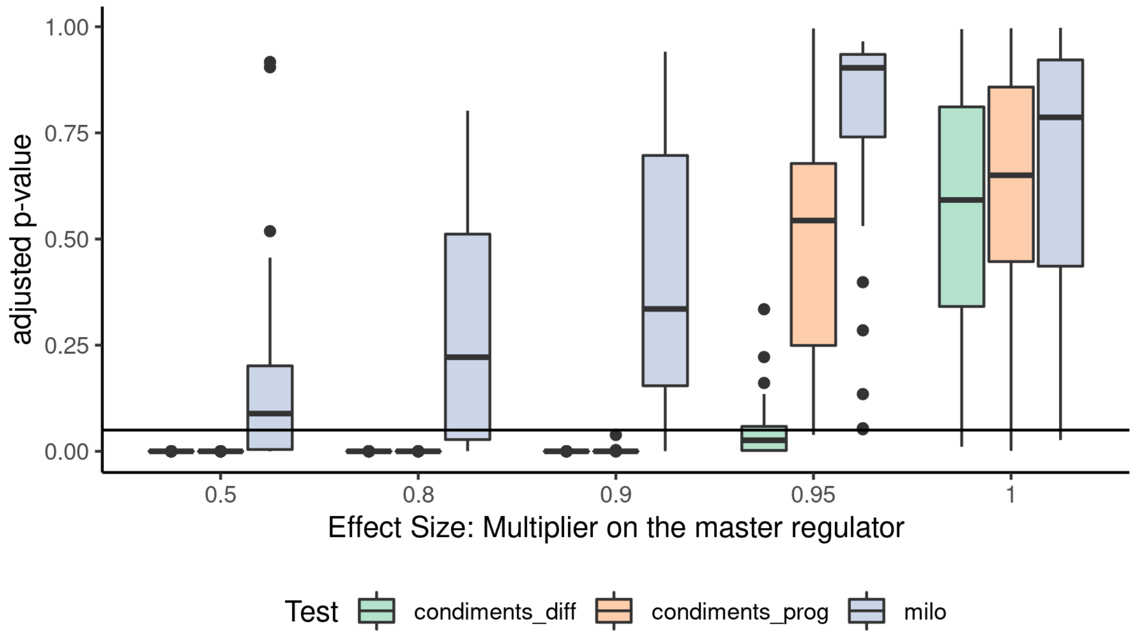

Figure S7: *Results on the third type of simulated dataset.* For all values of $m \in \{.5, .8, .9, .95, 1\}$, we generate null datasets with two lineages and three conditions and we compute the adjusted p-values of all tests that can handle 3 conditions. The distributions of p-values are then displayed. $m = 1$ is negative (no effect), while $m < 1$ is positive (some effect) with smaller values (toward the left) representing stronger effect.

S-2.4 Statistical validity and properties of the p-values of Step 3

To be valid, under the null, the empirical distribution of the J p-values p_j should verify the following, for all $p \in \{0 : 1\}$,

$$\frac{\sum_{j=1}^{J} 1_{p_j \leq p}}{J} \leq p.$$

We see below that this is true for all log-fold cut-offs. As expected, the test is more and more conservative as we increase the log-fold-change, ranging from mildly conservative for a log-fold-change of zero, to very conservative for a log-fold-change of 2.

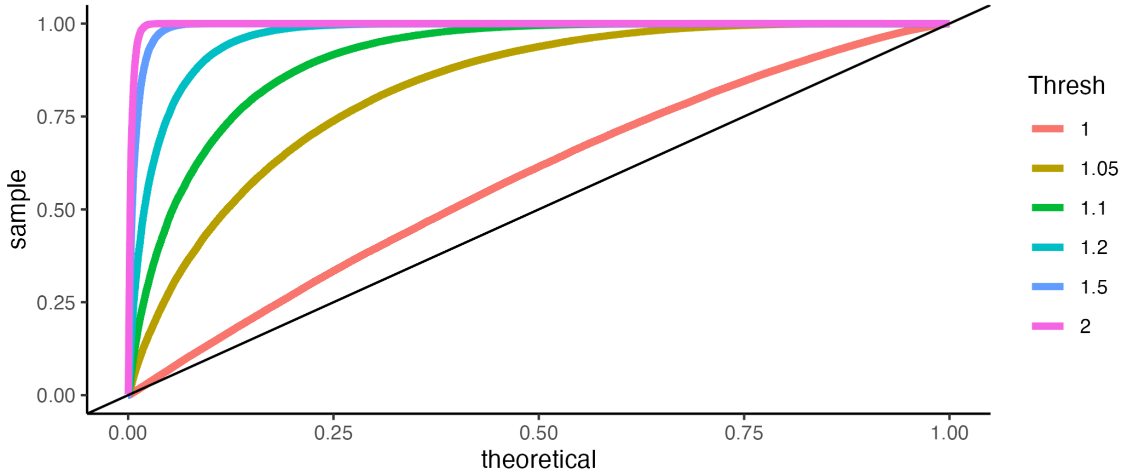

Figure S8: ***conditionTest** under the null.* Comparison of the distribution of p-values resulting from running the `conditionTest` for various log-fold-change cut-offs (*Thresh* in log scale), to the nominal uniform distribution.

We then focused on false discovery rate control. We adjusted the p-values from the `conditionTest` via BH-correction and computed the false discovery rate at varying levels of nominal control. We see that, when testing against a null of no change (log-fold-change of 0), `conditionTest` with BH-correction does not properly control the FDR at any level. On the other hand, when testing against a log-fold-change threshold of log(1.2) or greater, we control the nominal false discovery rate at appropriate levels for all values.

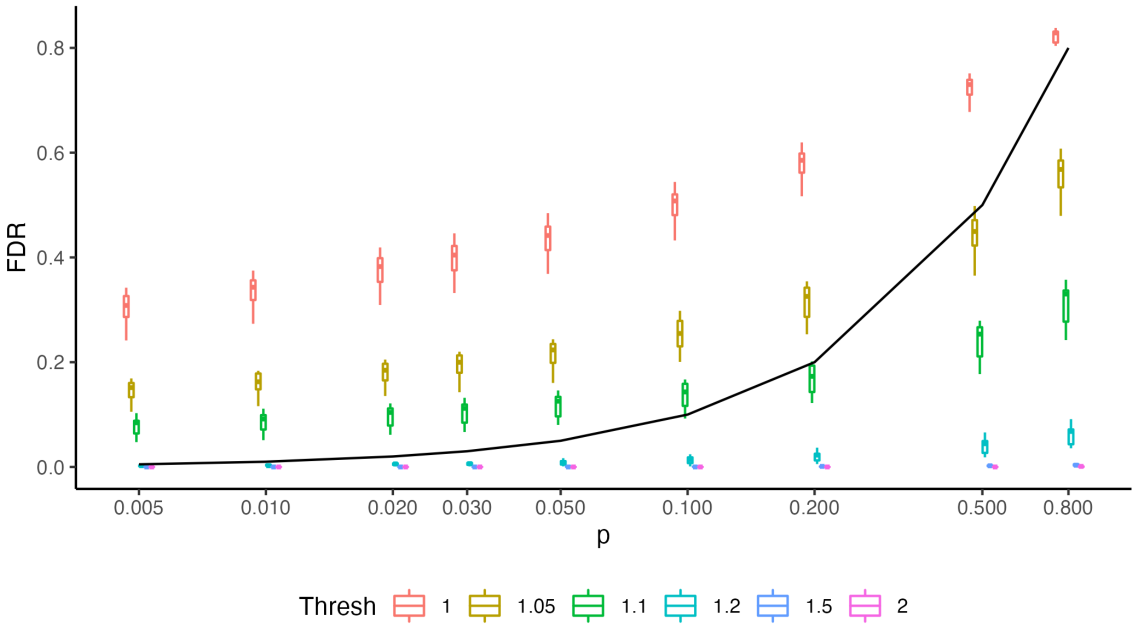

Figure S9: ***conditionTest** and FDR control.* Comparison of the true and nominal rates of false discoveries.

S-2.5 TGF-B

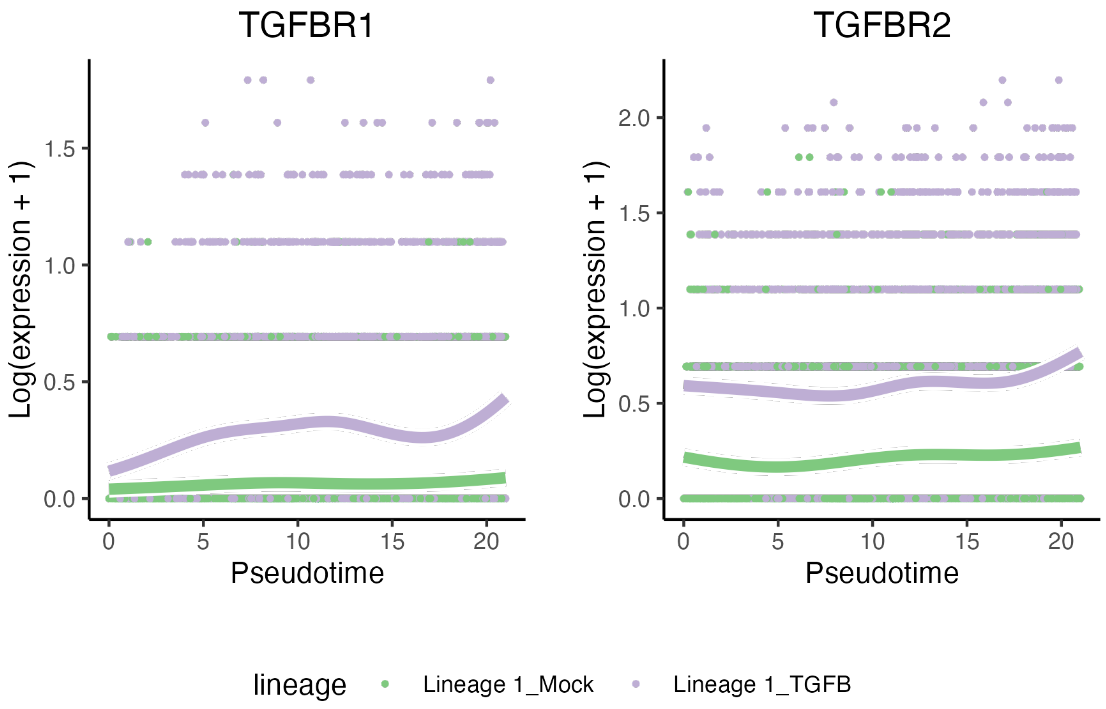

Figure S10: ***TGF-β** dataset: TGF-β receptors.* We fit smoothers for the two TGF-β receptors, **TGFBR1** and **TGFBR2**. Those two genes are more highly expressed in the TGF-β condition.

Table S1: *Gene Set Enrichment Analysis.* We perform a gene set enrichment analysis on all 10,100 genes expressed in the **Fibrosis** dataset, using the test statisic from the `patternTest` and `fgsea` [32]. We adjust for multiple testing by controlling the false discovery rate at the 5% level

pathway	pvalue	adjusted pvalue
Biological Adhesion	0.00002	0.0174885
Cell Population Proliferation	0.00002	0.0174885
Defense Response	0.00002	0.0174885
Negative Regulation Of Multicellular Organismal Process	0.00002	0.0174885
Anatomical Structure Formation Involved In Morphogenesis	0.00004	0.0174885
Animal Organ Morphogenesis	0.00004	0.0174885
Cell Activation	0.00004	0.0174885
Epithelium Development	0.00004	0.0174885
Reproduction	0.00004	0.0174885
Cell Cell Adhesion	0.00006	0.0196746
Locomotion	0.00006	0.0196746
Regulation Of Immune System Process	0.00006	0.0196746
Cell Migration	0.00008	0.0224853
Multi Organism Process	0.00008	0.0224853
Response To Corticosteroid	0.00010	0.0262328
Positive Regulation Of Immune System Process	0.00012	0.0277759
Tube Development	0.00012	0.0277759
Tube Morphogenesis	0.00016	0.0349771
Regulation Of Proteolysis	0.00020	0.0414202
Regulation Of Peptidase Activity	0.00024	0.0465036
Response To Organic Cyclic Compound	0.00026	0.0465036
Response To Oxygen Containing Compound	0.00026	0.0465036
Regulation Of Multicellular Organismal Development	0.00028	0.0479034

S-2.6 Fibrosis

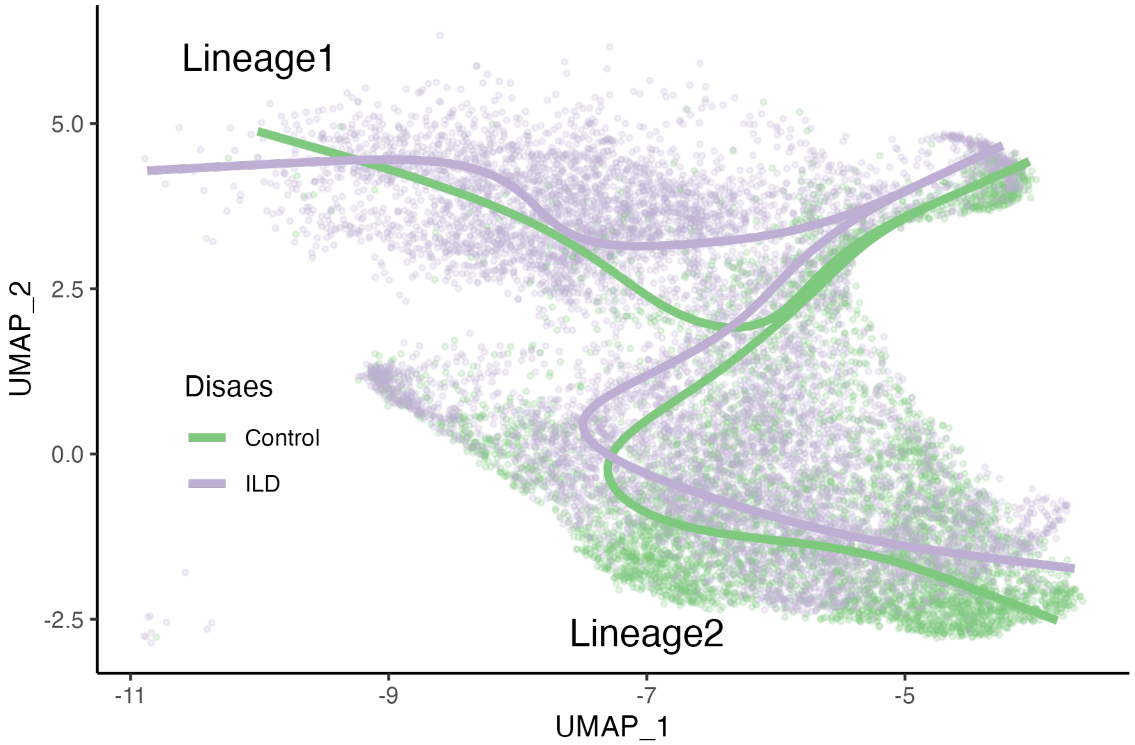

Figure S11: ***Fibrosis** dataset: fitting separate trajectories.* One trajectory is fitted per condition, using Slingshot. The absence of any cells from SCGB3A2+ in the control leads to an inappropriate shrinkage of the lineages (green curves) towards the AT1 cells.

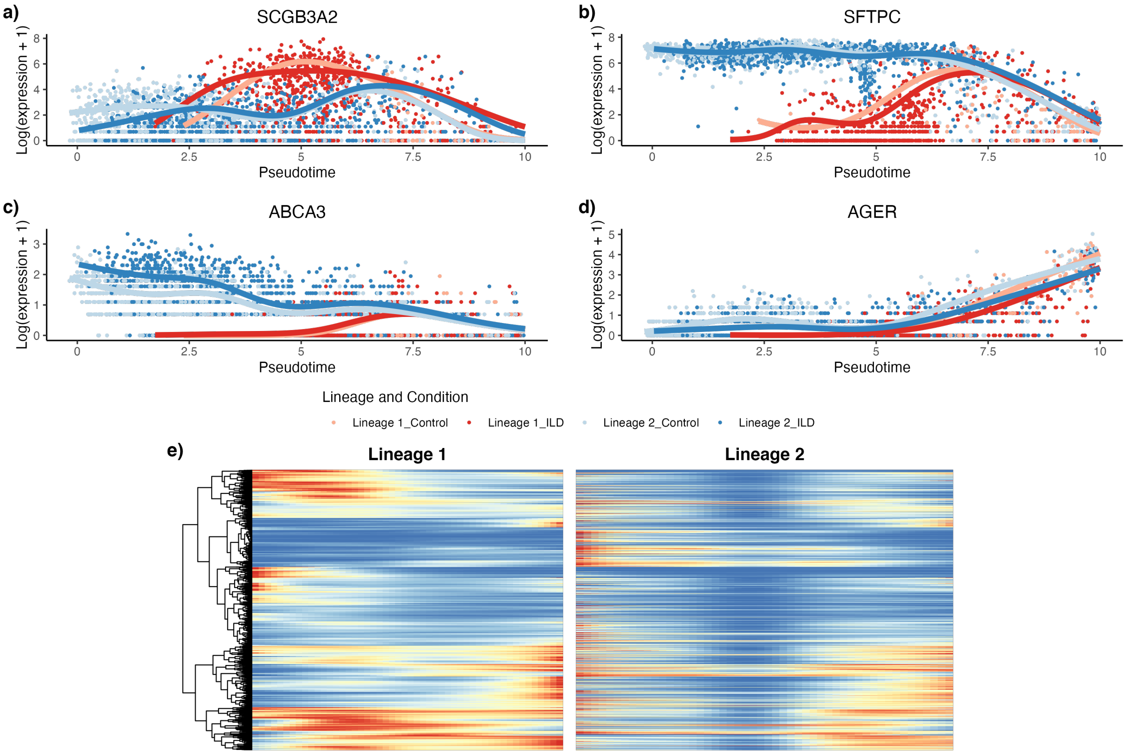

Figure S12: ***Fibrosis** dataset: Differential gene expression.* The tradeSeq gene expression model is fitted using the trajectory computed with slingshot. Differential expression between lineages is assessed using the `patternTest`. The four genes mentioned in the original paper all display differences between the two lineages, but none between the conditions **(a-d)**. After adjusting the p-values to control the FDR at a nominal level of 5%, we display genes in both conditions using a pseudocolor image **(e)**.

S-2.7 TCDD

Nault et al. [10] collected a dataset of 16,015 single nuclei to assess the hepatic effects of 2,3,7,8-tetrachlorodibenzo-p-dioxin or TCDD. In particular, they focused on the effect of TCDD on the 9,951 hepatocytes cells along the central-portal axis. This dataset is not a developmental dataset per se but still exhibits continuous changes along a spatial axis, demonstrating the versatility of the trajectory inference framework in general, and of the `condiments` workflow in particular.

Fig. S13a shows a reduced-dimensional representation of the dataset, with cells labelled according to treatment/control condition, while Fig. S13b shows the same plot colored by cell type, as derived by the authors of the original publication. The cells are aligned in a continuum, from central to mid-central and then mid-portal and portal. The imbalance score shows some spatial pattern (Fig. S13c). However, the nominal p-value associated with the `topologyTest` is .07. We therefore fail to reject the null and we infer a common trajectory using `slingshot` on the spatial clusters. This results in a single-lineage trajectory that respects the ordering of the spatial clusters (Fig. S13d). Note that, since the trajectory reflects a spatial continuum rather than a temporal one, the start of the trajectory is arbitrary. However, inverting the start and end clusters amounts to an affine transformation of the pseudotimes for all the cells. Step 2 and 3 are fully invariant to this transformation, so we can pick the Central cluster as the start of the trajectory.

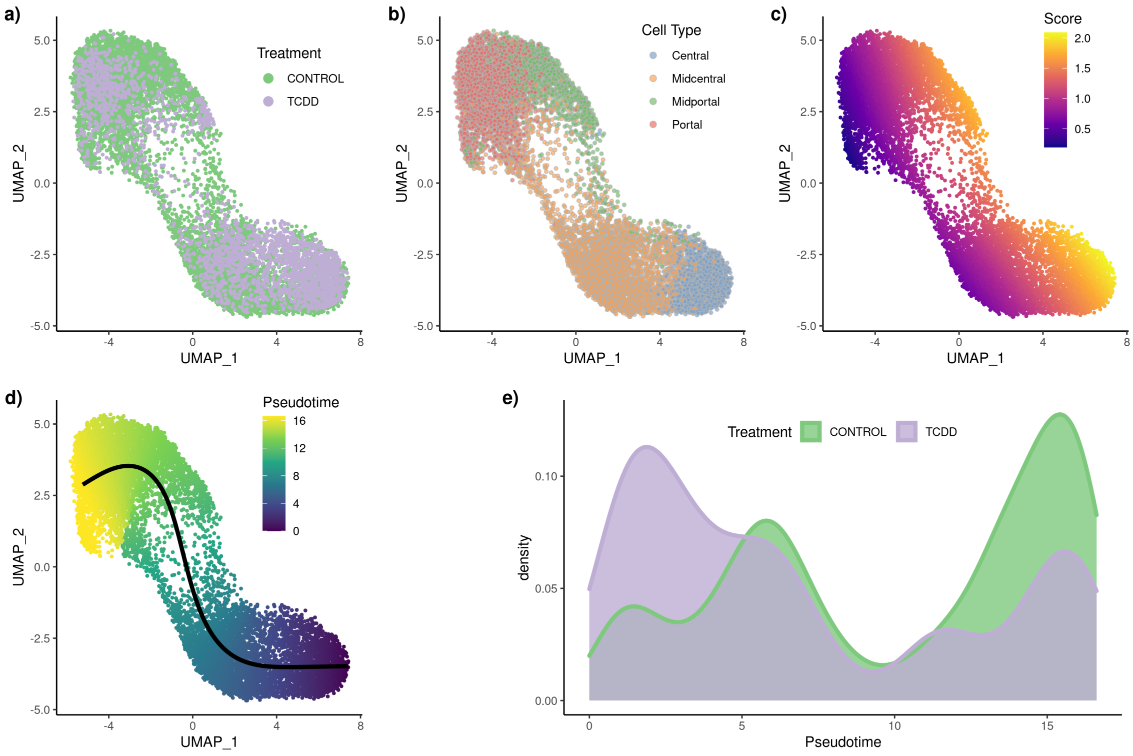

Figure S13: ***TCDD** dataset: Differential topology and differential progression.* After normalization and projection on a reduced-dimensional space, the cells can be represented, colored either by treatment label **(a)** or cell type **(b)**. Using the treatment label and the reduced-dimensional coordinates, an imbalance score is computed and displayed **(c)**. The `diffTopoTest` rejects the null and separate trajectories are fitted for each condition **(d)**. After mapping the lineages, there is also differential progression: the pseudotime distribution along the trajectory are not identical **(e)** and we indeed reject the null using the `diffProgressionTest`.

The densities of the treatment and control pseudotime distributions differ greatly visually (Fig. S13e), with the TCDD density heavily skewed toward the start of the trajectory. Indeed, the `progressionTest` has a nominal p-value $\leq 2.2 \times 10^{-16}$. This coincides with the finding of the original publication which highlighted the periportal hepatotoxicity of TCDD.

The ability of the `progressionTest` to correctly find large-scale changes in the spatial distribution of cells between conditions underscores why we favor fitting a common trajectory. Indeed, the p-value of

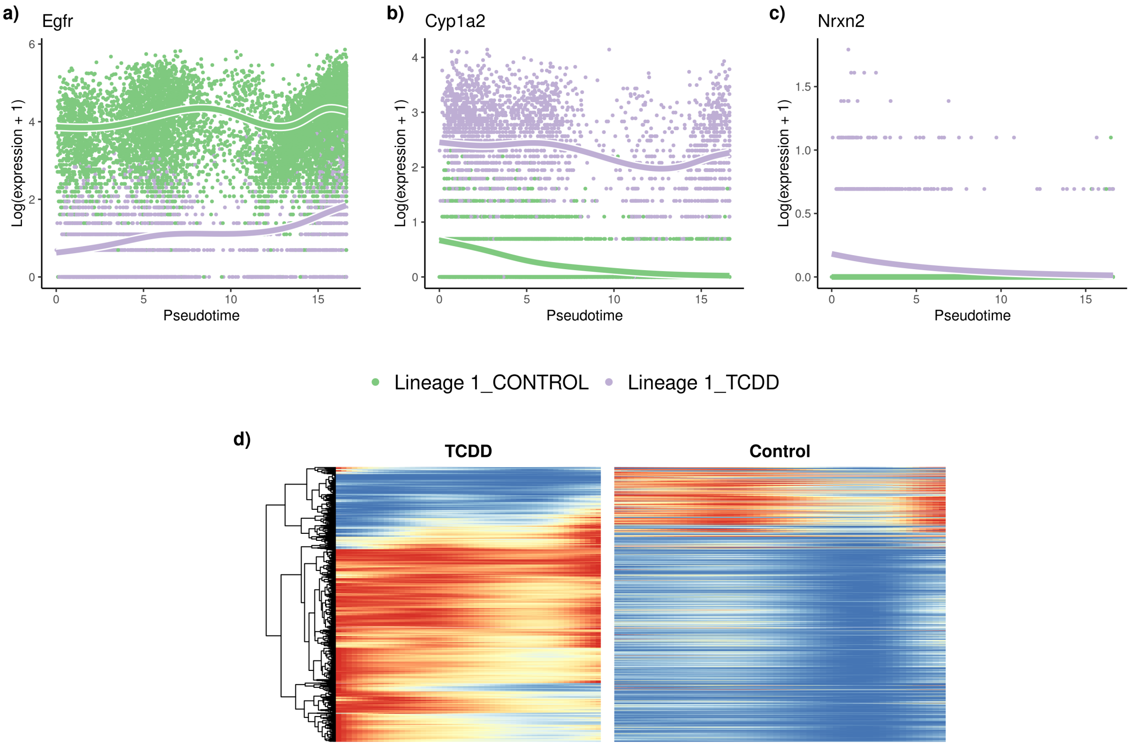

Figure S14: ***TCDD** dataset: Differential expression.* The tradeSeq gene expression model is fitted using the trajectory computed with slingshot. Differential expression between conditions is assessed using the `conditionTest` and genes are ranked according to the test statistics. The genes with the highest **(a)**, second highest **(b)**, and smallest **(c)** test statistics are displayed. After adjusting the p-values to control the FDR at a nominal level of 5%, we display genes in both conditions using a pseudocolor image **(d)**.

the `topologyTest` in Step 1 is rather small and would have been below .05 if we had not conducted a test against a threshold. However, testing against a threshold and thus fitting a common trajectory does not stop the workflow from finding large-scale differences between conditions in Step 2 and results in a more stable estimate of the trajectory.

After gene filtering, we test 8,027 genes for spatial differential expression between conditions and we find 2,114 DE genes when controlling the FDR at a nominal level of 5%. The genes with the largest, second largest, and smallest test statistics are displayed in Fig.S14a-c. Similarly to Nault et al. [10], we obtain a list of zonal genes from Halpern et al. [49]. The proportion of zonal genes among the DE genes is twice their proportion among non-DE genes.

S-2.8 KRAS

KRAS dataset

Xue et al. [27] studied the impact of KRAS(G12C) inhibitors at the single-cell level on three models of KRAS(G12C) lung cancers. Specifically, they examined how various cell populations react to these inhibitors and how some cells can return in proliferation mode shortly after the end of the treatment. Here, we want to investigate how the three cancer models (H358, H2122, and SW1573) differ in their response to the KRAS(G12C) inhibitors.

We use the reduced-dimensional representation from the original paper to display the 10,177 cells from the various types (Fig S15a). Using the cancer type labels and the reduced-dimensional coordinates, an imbalance score can be computed (Fig S15b); some regions clearly show an imbalance. This is further confirmed by the `topologyTest`, with p-value smaller than 2.2×10^{-16}. We therefore do not fit a common trajectory to all cancer types (Fig S15c).

Note that this does not necessarily imply that the trajectory of reaction to the KRAS(G12C) inhibitors is different between cancer types. Indeed, this may also reflect strong batch effects between conditions, which the normalization scheme was unable to fully remove when integrating the three cancer types in

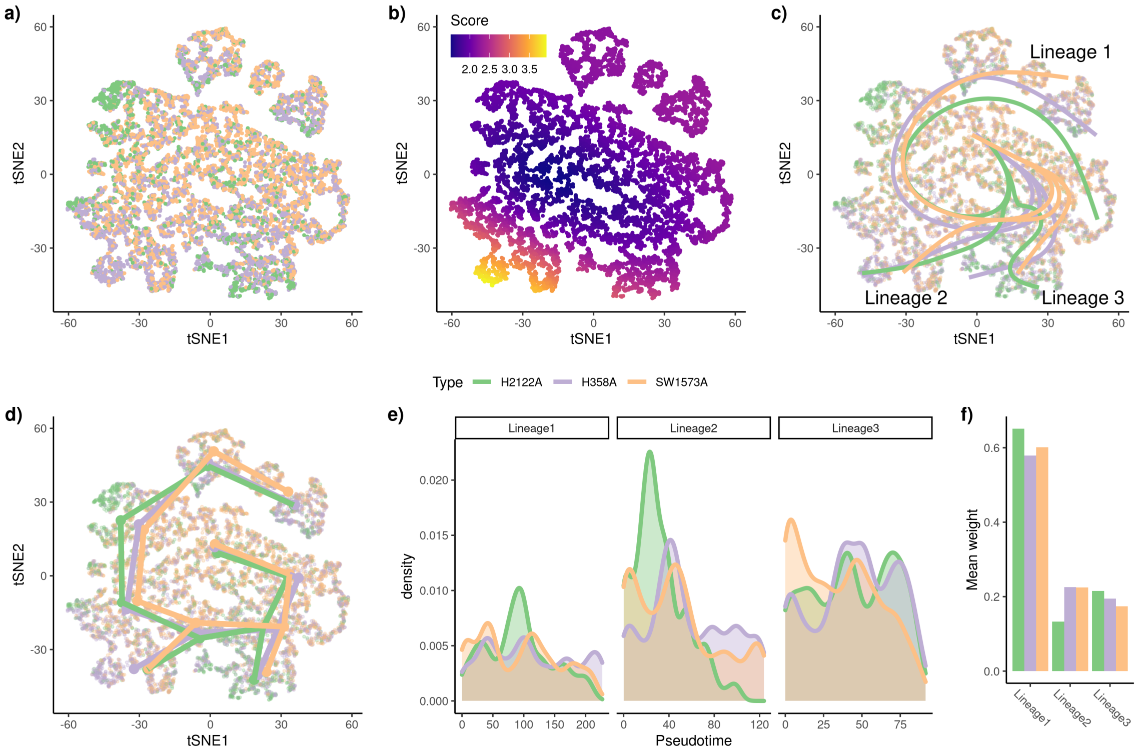

Figure S15: ***KRAS*** *dataset: Differential topology, differential progression, and differential fate selection.* Using the reduced-dimensional representation of the original publication (t-SNE), the cells can be colored by cancer type (a.). Using the cancer type label and the reduced-dimensional coordinates, an imbalance score is computed and displayed **(b)**. The `topologyTest` rejects the null hypothesis of a common trajectory, we thus fit one trajectory per condition **(c)**. However, the skeleton graphs have the same structure **(d)**, so we can progress to the next steps in the `condiments` workflow. There is differential progression **(e)** and we indeed reject the null of identical pseudotime distributions along the trajectory using the `progressionTest`. Similarly, there is differential fate selection **(f)** and we reject the null of identical weight distributions along the trajectory using the `fateSelectionTest`. Here, we summarize the distributions by looking at the average weight for each lineage in each condition, which already shows some clear differences.

one common reduced-dimensional representation. Thus, it is not really possible to draw a biological conclusion at this first step. However, this does mean that a separate trajectory should be fitted to each condition.

Here, the trajectories, although different, are similar enough that we can still use an underlying common skeleton (Fig S15d). Indeed, we keep the tree structure derived by computing the minimum spanning tree (MST) on the clusters using all cells. This way, it is possible to derive a one-to-one mapping between the lineages of the three trajectories and we respect the assumptions detailed in Section 1.2 that are necessary for the `progressionTest` and `fateSelectionTest`.

Using this common mapping, we can then proceed to the `progressionTest`. At the global trajectory level, the nominal p-value is smaller than 2.2×10^{-16}, showing clear differential progression. At the lineage level, all three lineages show strong differential progression, with p-values of 2.2×10^{-16}, 1.2×10^{-12}, and 1.2×10^{-14}, respectively. The density plots for the pseudotime distributions at the single-lineage level (Fig S15e) indicate that the differential progression is driven by a group of cells from cancer type H2122A. This matches the top left part of the reduced-dimensional plot, the region where cells exit the initial inhibition stage to enter the reactivation stage. The second lineage also shows a difference between H2122A and the two other models. The pseudotime distribution is heavily skewed toward earlier points in that model compared to the other two. Lineage 2 represents differential progression to a drug-induced state. In Lineage 3, it is the SW1573A model that displays more differential progression.

The `fateSelectionTest` also has a p-value smaller than 2.2×10^{-16}. Although all pairwise comparisons are significant, the test statistics are much higher for the Lineage 2 vs. 1 and Lineage 2 vs. 3

comparisons. This again suggests that one model differentiates less into the drug-induced path, compared to the other two. Since the weights have to sum to 1, the 3-dimensional distribution can be fully summarized by any two components. Fig S16 shows clear differences in distributions but visually interpreting different 2D distributions is still challenging. A simpler way to compare the distributions is to look at the average weight in each condition for each lineage (Fig S15f). This ignores the correlation between lineages but still allows for some interpretation. We can see in particular that Lineages 1 and 3 have greater weights for H2122A than for the other two conditions, which is consistent with the different pairwise statistics.

With the mapped trajectories, we can also perform gene-level analysis using the `conditionTest`. When comparing genes across all lineages and conditions, we find 363 differentially expressed genes when controlling the FDR at nominal level 5%. We show the genes with the highest, second highest, and smallest test statistics in Fig. S17a-c. Displaying these global patterns across all three lineages and all three conditions makes it hard to interpret. We therefore focus on the first (and longest) lineage. In that lineage, we find 366 DE genes and we show their expression patterns along Lineage 1 in all three cancer models in Fig. S17d.

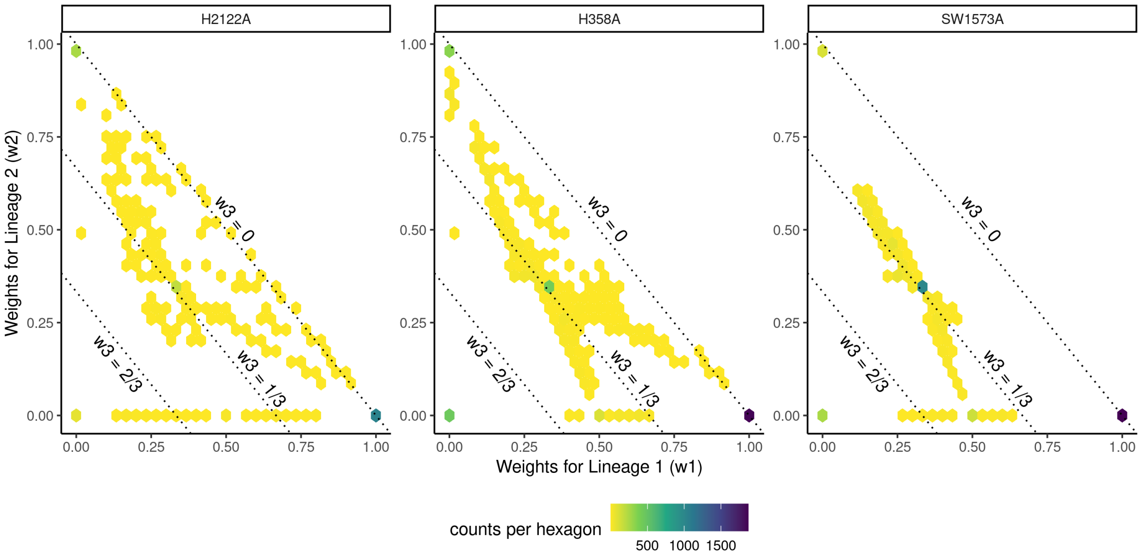

Figure S16: ***KRAS*** *dataset: Differential fate selection.*

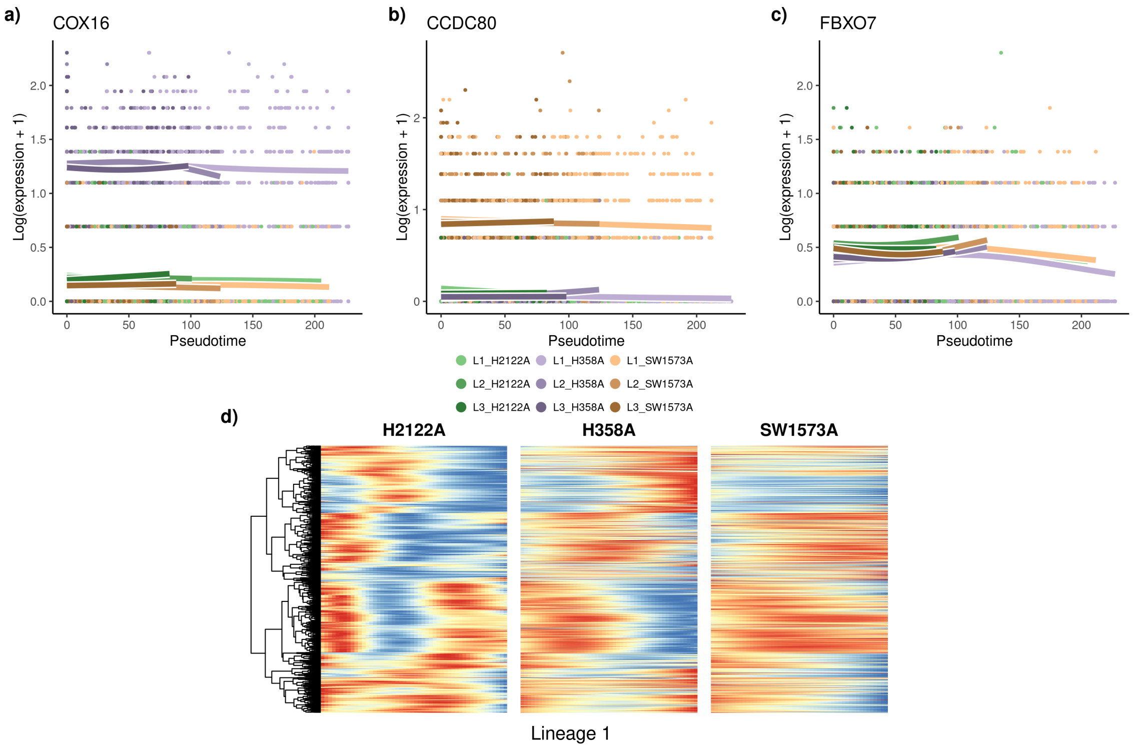

Figure S17: ***KRAS*** *dataset: Differential expression.* The tradeSeq gene expression model is fitted using the trajectory computed with slingshot. Differential expression between conditions is assessed using the `conditionTest` and genes are ranked according to the test statistics. The genes with the highest **(a)**, second highest **(b)**, and smallest **(c)** test statistics are displayed. Focusing on the first lineage, we select all differentially expressed genes in that lineage after adjusting the p-values to control the FDR at a nominal level of 5%. We display the genes for all three conditions using a pseudocolor image **(d)** along this first lineage.
